# Formation of Raft-Like Assemblies within Clusters of Influenza Hemagglutinin Observed by MD Simulations

**DOI:** 10.1371/journal.pcbi.1003034

**Published:** 2013-04-11

**Authors:** Daniel L. Parton, Alex Tek, Marc Baaden, Mark S. P. Sansom

**Affiliations:** 1 Department of Biochemistry, University of Oxford, Oxford, United Kingdom; 2 Institut de Biologie Physico-Chimique, Centre National de la Recherche Scientifique, UPR9080, Université Paris Diderot, Sorbonne Paris Cité, Paris, France; 3 Université Pierre et Marie Curie, UPMC-Sorbonne Universités, Paris, France; Max Planck Institute for Biophysical Chemistry, Germany

## Abstract

The association of hemagglutinin (HA) with lipid rafts in the plasma membrane is an important feature of the assembly process of influenza virus A. Lipid rafts are thought to be small, fluctuating patches of membrane enriched in saturated phospholipids, sphingolipids, cholesterol and certain types of protein. However, raft-associating transmembrane (TM) proteins generally partition into Ld domains in model membranes, which are enriched in unsaturated lipids and depleted in saturated lipids and cholesterol. The reason for this apparent disparity in behavior is unclear, but model membranes differ from the plasma membrane in a number of ways. In particular, the higher protein concentration in the plasma membrane may influence the partitioning of membrane proteins for rafts. To investigate the effect of high local protein concentration, we have conducted coarse-grained molecular dynamics (CG MD) simulations of HA clusters in domain-forming bilayers. During the simulations, we observed a continuous increase in the proportion of raft-type lipids (saturated phospholipids and cholesterol) within the area of membrane spanned by the protein cluster. Lateral diffusion of unsaturated lipids was significantly attenuated within the cluster, while saturated lipids were relatively unaffected. On this basis, we suggest a possible explanation for the change in lipid distribution, namely that steric crowding by the slow-diffusing proteins increases the chemical potential for unsaturated lipids within the cluster region. We therefore suggest that a local aggregation of HA can be sufficient to drive association of the protein with raft-type lipids. This may also represent a general mechanism for the targeting of TM proteins to rafts in the plasma membrane, which is of functional importance in a wide range of cellular processes.

## Author Summary

The cell membrane is composed of a wide variety of lipids and proteins. Until recently, these were thought to be mixed evenly, but we now have evidence of the existence of “lipid rafts” — small, slow-moving areas of membrane in which certain types of lipid and protein accumulate. Rafts have many important biological functions in healthy cells, but also play a role in the assembly of influenza virus. For example, after the viral protein hemagglutinin is made inside the host cell, it accumulates in rafts. Exiting virus particles then take these portions of cell membrane with them as they leave the host cell. However, the mechanism by which proteins associate with lipid rafts is unclear. Here, we have used computers to simulate lipid membranes containing hemagglutinin. The simulations allow us to look in detail at the motions and interactions of individual proteins and lipids. We found that clusters of proteins altered the properties of nearby lipids, leading to accumulation of raft-type lipids. It therefore appears that aggregation of hemagglutinin may be enough to drive its association with rafts. This helps us to better understand both the influenza assembly process and the properties of lipid rafts.

## Introduction

The interplay between membrane lipids and proteins plays a key role in a number of cellular processes [1], [2] including the replication and release of viruses. For example, in the latter stages of the replication cycle of influenza virus A, the viral genome and associated proteins gather at the plasma membrane, from where they bud via exocytosis. The released virion is thus surrounded by a lipid envelope, which incorporates three types of transmembrane (TM) protein: the two spike proteins, HA and neuraminidase (NA), and the M2 channel. The envelope is characterized by a high concentration of spike proteins (ca. 8000 µm^−2^ [3]), and a distinct lipid composition. Compared with the host cell membrane, the envelope is enriched in sphingolipids and cholesterol, and depleted in glycerophospholipids [4]. These features have been suggested to originate from the association of HA and NA with putative lipid rafts in the plasma membrane, prior to viral budding [5]–[8].

Lipid rafts can be generally described as small (<100 nm diameter), fluctuating patches of membrane enriched in saturated phospholipids, sphingolipids, cholesterol and certain types of protein, including most GPI-anchored and acylated proteins, and some TM proteins. They are known to have importance in membrane signaling and trafficking [2]. However, their exact nature has been subject to discussion, particularly due to the difficulties of direct visualization *in vivo* [2].

Early evidence for the association of HA with lipid rafts arose from its presence in detergent-resistant membranes extracted from the plasma membranes of influenza-infected cells [9], [10]. More recently, fluorescence resonance energy transfer (FRET) studies in live cells have indicated the association of full-length HA with raft-markers (acylated proteins) in the plasma membrane intracellular (IC) leaflet [11], and the association of a fragment of HA containing the TM and cytoplasmic regions with raft-markers (GPI-anchored proteins) in the extracellular (EC) leaflet [12]. These studies also highlight how raft association can be influenced by palmitoylation of HA at residues in the cytoplasmic domain, and by mutation of hydrophobic amino acids towards the EC side of the TM domain. Other studies have provided direct visualization of clusters of HA in the plasma membrane via immunogold-labeling electron microscopy (EM) [6], [13], and in live fibroblasts via fluorescence photoactivation localization microscopy (FPALM) [14], albeit without direct evidence of raft-association.

Rafts have been compared with liquid-ordered (Lo) domains in model lipid bilayers. Lo domains generally form on larger length-scales than those of *in vivo* rafts, but have been employed in many experiments as model raft systems [2]. An archetypal domain-forming model membrane comprises a ternary mixture of saturated phospholipid (often phosphatidylcholine (PC) or sphingomyelin), unsaturated phospholipid and cholesterol, which will undergo spontaneous temperature-dependent separation into Lo (enriched in saturated lipids and cholesterol) and Ld (liquid-disordered; enriched in unsaturated lipids) domains [15]. Lateral phase segregation is thought to be driven primarily by the preference of cholesterol for association with saturated lipid tails, which can adopt a favorable ordered conformation when adjacent to the rigid, planar sterol ring [16]. The Lo phase is therefore distinguishable from the Ld phase by increased phospholipid tail ordering, but (unlike the solid-ordered S_o_ (gel) phase) without a drastic decrease in lateral mobility; the lateral diffusion coefficient is reduced by a factor of ∼2–3 [17].

A number of important differences separate the behavior of TM proteins in domain-forming model membranes from their behavior in lipid rafts in plasma membranes. Notably, raft-associating TM proteins partition into the Ld domain in model membranes, rather than the Lo domain as might be expected for a true raft-mimic [18]–[22]. Possible explanations for this apparent disparity in behavior are the much higher protein concentration present in the plasma membrane (up to 60% dry mass [23]), and interactions with cytoskeletal components.

Experimental approaches to date have not permitted direct observation of the interactions of TM proteins with lipid rafts. Molecular dynamics (MD) simulations of membrane proteins [24] have been used in a number of studies to investigate domain-forming membranes in atomistic detail [25]–[27]. Coarse-grained (CG) force fields [28]–[33] allow longer length and time scales to be addressed than do more conventional atomistic simulations. Such CG simulations can reproduce the domain-forming properties of model membranes composed of ternary lipid mixtures [34]. Related studies have investigated concerted lipid diffusion within domains [26], the influence of lipid domain properties and leaflet asymmetry on inter-leaflet coupling [35], and the partitioning of simple TM peptides and peripheral membrane proteins in domain-forming membranes [36]–[39]. A related CG simulation technique, dissipative particle dynamics (DPD), has been used to investigate the effect of acylation on the tilt angle of a model TM protein [40].

In the current study, we use CG MD simulations to investigate the molecular details of the interactions between HA and raft-type lipids in domain-forming membranes. By including HA within a membrane at a high concentration, the simulations address a limitation of some experimental studies, namely the difficulty of incorporating proteins into model membranes at high concentrations [41]. The overall membrane protein concentration in the simulations (ca. 4000 µm^−2^) is comparable to that expected of a typical cell membrane or in an influenza virus (ca. 8000 µm^−2^) [3]. The simulations show that raft-type lipids are enriched within dynamic nanoclusters of HA proteins within the membrane. We therefore suggest that a high local concentration of HA may be sufficient for association of the protein with rafts in the plasma membrane.

## Results

### CG MD simulations of HA in domain-forming membranes

**Figure 1:**
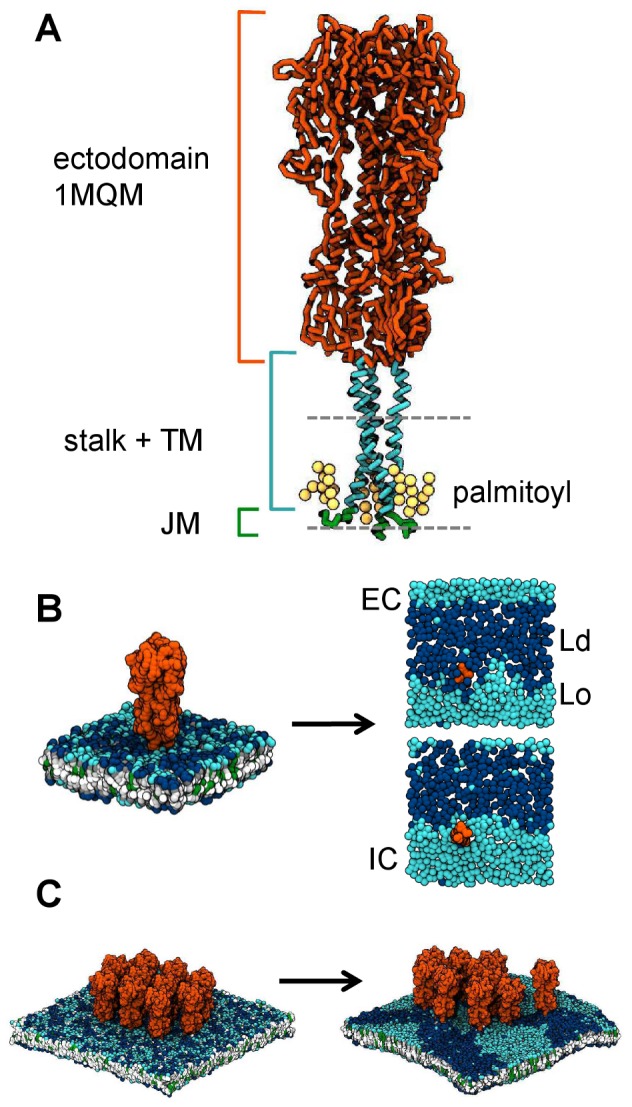
CG model of HA, simulated in domain-forming membranes with varying local concentration. **A** The CG model of HA. The ectodomain (orange) was derived from the X-ray structure (PDB code: 1MQM) (55). The stalk and TM domain (cyan) were modeled as α-helix. The C-terminal tail (green) was modeled as an unstructured region with attached palmitoyl tails (yellow). The gray broken lines indicate the approximate location of the lipid bilayer. **B** Snapshots of the beginning and end of one of the two 12 µs *1HA* simulations. The protein is shown in orange, DPPC headgroups in light blue, DLiPC headgroups in dark blue, phospholipid tails in gray and white, and cholesterol in green. The t = 12 µs snapshots (with the EC leaflet shown in the upper right image, and the IC leaflet in the lower right image) show only the protein TM domain and lipid phosphate particles. Ld domains are composed primarily of DLiPC (dark blue). Lo domains are composed primarily of DPPC (light blue) and cholesterol (which is not shown for clarity, but is generally associated with the same regions as DPPC). C Snapshots of the beginning and end of one of the four 12 µs 10HA simulations. A slight positive membrane curvature can be observed in the final snapshot.

A CG model of HA (Fig. 1A) was built as described in the Methods. The protein was simulated in three different membrane environments (Table 1): as a single membrane protein in a mixed lipid bilayer (simulation *1HA*); as a single protein in a pure DLiPC bilayer (*1HA-DLiPC*); and as a cluster of ten membrane proteins in a mixed lipid bilayer (*10HA*). In all cases, the protein TM domain remained situated within the bilayer at the expected position. The short cytoplasmic tails (which were modeled as unstructured sequence) associated with the membrane-solvent interface, and were oriented roughly perpendicular to the TM domain. This orientation allowed the nine palmitoyl chains attached to each protein trimer to be incorporated into the IC (intracellular) leaflet of the bilayer. Proteins were able to tilt dynamically within the membrane; HA adopted an average tilt angle of 8°±5 relative to the bilayer normal in the *1HA* simulations.

**Table 1:** Simulations performed, with protein lateral diffusion coefficients (analyzed over the final 4 µs of each simulation).

Simulations	Membrane composition	Membrane dimensions (nm^2^)	Protein diffusion coeff. (×10^−8^ cm^2^ s^−1^)
*1HA-DLiPC*	DLiPC	20×20	3.5
*1HA* (×2)	0.35∶0.35∶0.3 DPPC/DLiPC/chol	20×20	1.1±0.1
*10HA* (×4)	0.35∶0.35∶0.3 DPPC/DLiPC/chol	50×50	0.73±0.15
*0HA*	0.35∶0.35∶0.3 DPPC/DLiPC/chol	50×50	-

DLiPC = dilinoleoylphosphatidylcholine (di-18:2-PC), chol = cholesterol. Error estimates for the diffusion coefficients represent the standard deviation between the values calculated from separate simulations.

To investigate the interactions of HA with lipid domains, the *1HA* and *10HA* simulations were conducted with membranes comprising a ternary mixture of saturated phospholipid, unsaturated phospholipid and cholesterol. Comparable lipid compositions have been employed in studies of lipid domains in model membranes [15], and in previous simulations of domain-forming membranes [34]. During the two 12 µs *1HA* simulations, the lipids became segregated into two domains, each taking up approximately half of the membrane area (Fig. 1B). One domain (Lo) was composed primarily of DPPC and cholesterol, and the other (Ld) of DLiPC; other physical properties such as tail ordering and lateral diffusion coefficients were consistent with their identification as Lo and Ld domains respectively (SI Fig. S1).

In contrast with previous simulations, which showed partitioning of (non-raft associating) TM peptides and proteins into Ld domains [36], [37], the single HA protein in the *1HA* simulations occupied a position at the *interface* between the two domains. Similar interfacial partitioning has been observed in MARTINI simulations of H-Ras (a raft-associating, lipid-anchored, peripheral membrane protein) [38] and palmitoylated WALP (a simple, model TM helix) [39], and in an AFM study of the raft-associating acylated protein N-Ras [42]. Experimental studies of HA have indicated partitioning into either the Ld or Lo domains, consistent with a lack of clear preference [22], [43].

The *1HA* simulations also revealed that the protein retained a short (∼2 lipids), exchanging annulus of DLiPC in the EC leaflet, and a similar annulus of DPPC and cholesterol in the IC leaflet (Fig. 1B). Thus, it seems likely that while the TM helix *per se* may exhibit a general affinity for unsaturated lipids (perhaps due to the irregularity of the protein surface), the palmitoyl chains attached to the IC tail of the protein preferentially interact with saturated lipids. These preferential interactions also affect the tendency for interleaflet registration of domains. This feature has been observed in MARTINI CG simulations with similar lipid compositions [34], although it has also been shown that increasing hydrophobic mismatch between Lo and Ld domains can result in antiregistration [35]. The lipid mixture used in these studies exhibits a strong tendency towards domain registration in the absence of other effects. In the *1HA* simulation, however, the preference of the protein for specific lipid annuli (resulting in antiregistered domains) overcomes any energetic saving from domain registration in the area immediately proximal to the protein (Fig. 1B). It is also feasible that the HA annuli may act as nucleation sites for domain formation. However, simulations of equivalent bilayers in the absence of protein did not suggest a difference in the kinetics of domain formation.

**Figure 2:**
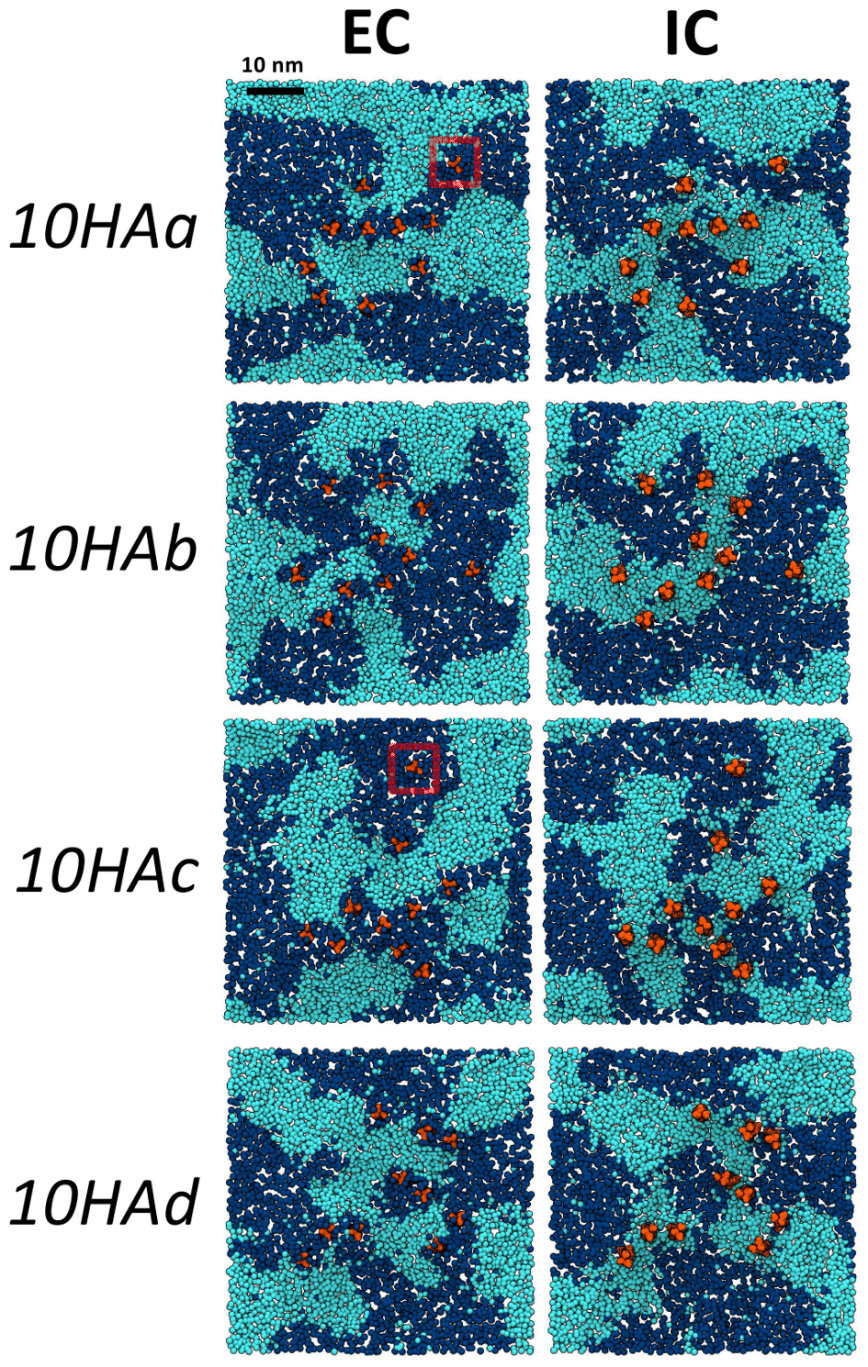
Simulations of HA clusters in domain-forming membranes. Top-down snapshots of the four *10HA* systems (labeled *10HAa-d*) after ∼12 µs of simulation. Protein TM domains are shown in orange, DPPC phosphates in light blue, and DLiPC phosphates in dark blue. Cholesterol, which displayed strong spatial correlation with DPPC throughout the simulations, is not shown for clarity. EC leaflets are shown in the left column, and IC leaflets in the right column. Protein clusters have been centered in the images shown (a graph of the protein paths during simulation b is shown in SI Fig. S2). All of the proteins aggregated via contacts between ectodomains (not shown for clarity), except those ringed in red, which remained unaggregated throughout the simulations.

The *10HA* simulations were designed to investigate how HA at a high local concentration interacts with lipid domains. Ten HA proteins were arranged close together in a regular array, and embedded in a preformed mixed lipid bilayer (Fig. 1C). Four replica systems were simulated for 12 µs each – snapshots are shown in Fig. 2, and a plot of the protein paths during one of the simulations is shown in Fig. S2. During the simulations, most proteins quickly aggregated by tilting and forming contacts via their ectodomains. Two cases were observed of proteins which remained unaggregated throughout the simulations (red circles in Fig. 2). Aggregated proteins were able to separate, but contacts were generally quickly reestablished with the same or other protein partners. Importantly, the bulky ectodomains prevented direct contacts between TM domains, and lipids were always present between any pair of proteins. Aggregation also reduced the lateral diffusion coefficient of the proteins compared to the *1HA* simulations (Table 1).

The proteins retained short, exchanging annuli of DLiPC in the EC leaflet, and of DPPC/cholesterol in the IC leaflet, as in the *1HA* simulations. Many proteins again appeared to be situated at domain interfaces, but domain topology was much more complex than in the *1HA* system. The tendency for domain registration was also strongly reduced within the vicinity of the protein clusters. By contrast, the *0HA* system – a 50×50 nm^2^ membrane of the same ternary lipid composition, without HA proteins – underwent phase separation into two distinct domains (with strong interleaflet registration) over the same timescale (see Fig. S3). The presence of the HA cluster thus appeared to be inhibiting the formation of large domains, which is likely due to the combination of the irregular shape of the HA cluster and the preference of individual proteins for partitioning at domain boundaries. This behavior is consistent with Ising and related models of two component lipid bilayers, which showed that the presence of immobilized membrane protein “obstacles” resulted in the formation of relatively small dynamic assemblies, rather than extended domains [44].

### Enrichment of raft-type lipids within HA clusters

**Figure 3:**
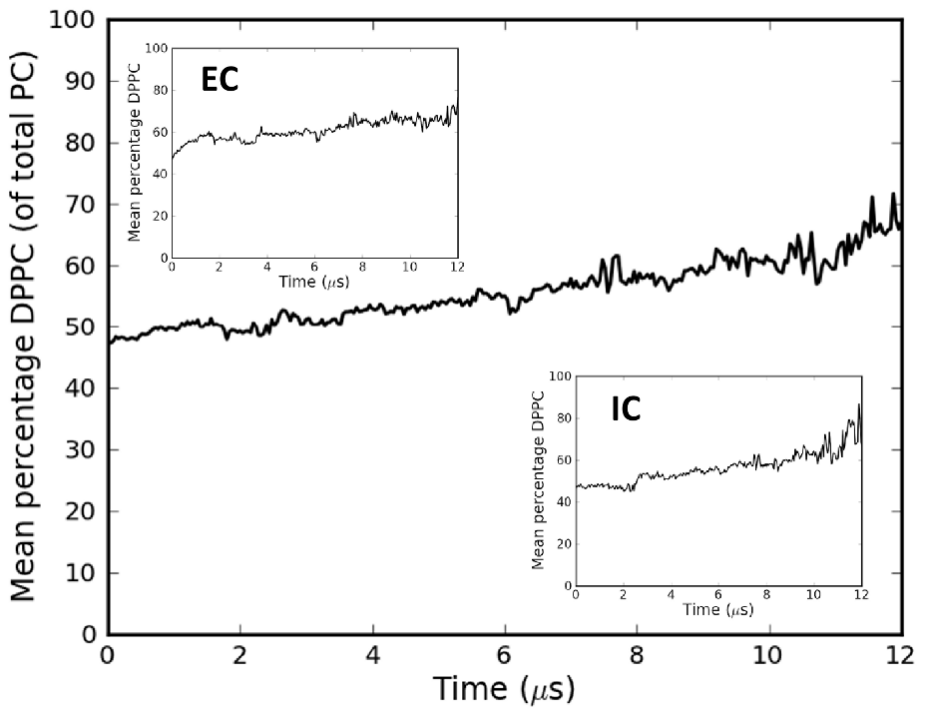
Lipid composition within the HA clusters. DPPC composition (as a percentage of total PC) within the HA clusters averaged across the four *10HA* simulations. The main graph shows the analysis for both leaflets of the bilayer combined, while the inset graphs show analysis of the individual leaflets. Details of the algorithm for defining the cluster interior are included in the Supporting Information.

The total membrane area in the *10HA* simulations (50×50 nm^2^) was set to be substantially larger than the area of the protein patch (ca. 20×20 nm^2^) so as to allow a clear distinction between lipids within the protein patch and bulk membrane. Analysis of the evolution of lipid composition within the protein clusters over time (Fig. 3) indicated a general increase in the fraction of DPPC (see Supporting Information for details of the definition of the cluster interior). This trend was observed in both leaflets of the membrane. Cholesterol also displayed an increase in concentration within the cluster (SI Fig. S4), reflecting the strong spatial correlation between these two lipids.

Analysis of diffusion coefficients during the *1HA* simulations (measured over the timescale 8–12 µs) showed that DLiPC diffusion was strongly attenuated by proximity to the protein, while DPPC was relatively unaffected (SI Fig. S1). A number of previous simulation studies have reported a reduction in lipid diffusion adjacent to TM proteins [25].

**Figure 4:**
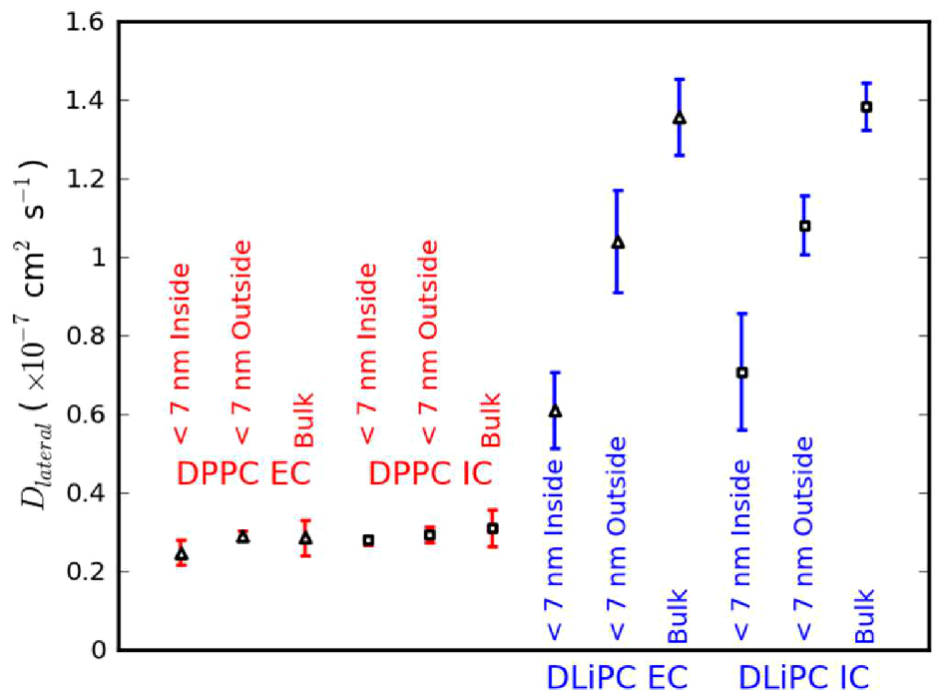
Lipid lateral diffusion coefficients. Diffusion coefficients were calculated individually for each of the four *10HA* simulations, then averaged (error bars represent one standard deviation). Lipids were analyzed in three regions: <7 nm from the nearest protein and inside the cluster (according to the algorithm described in the Supporting Information); <7 nm from the nearest protein and outside the cluster; and bulk membrane (defined as >7 nm from the nearest protein and outside the cluster). Displacements *r* were measured using timesteps ranging from 4 to 400 ns, and converted to mean square displacements, <*r* ^2^>, by averaging over each lipid (within the relevant region), and over the final 4 µs of simulation. In each case, the timestep displaying a mean displacement <r> of 2 nm was used to calculate the diffusion coefficient, to avoid measurement of fast, subdiffusive lipid motion which takes place over shorter length scales. As discussed in the main text, lipids within the cluster interiors undergo only subdiffusive motion (due to the crowding effects of the proteins) over the duration of the simulations, and their diffusion coefficients should therefore not be directly compared with those from other studies.

Lipid diffusion coefficients for the *10HA* simulations (Fig. 4) were analyzed in three different regions: lipids within the cluster; lipids outside the cluster; and bulk lipids. This revealed that diffusion of DLiPC within the cluster is considerably slower than that of DLiPC outside the cluster and even more so than that of bulk DLiPC. In contrast, only a small effect is seen for DPPC. It therefore appears that the decrease in DLiPC diffusion coefficient in proximity to the protein is amplified by increased local protein concentration, while the smaller effect on DPPC remains relatively insignificant. Lipid tail ordering was also analyzed, and found to be essentially unaffected by position inside or outside the cluster (data not shown).

The attenuation in lipid diffusion within the protein cluster arises from steric crowding by the slow-diffusing proteins. This implies an increase in the chemical potential of lipids within the protein cluster, relative to those in the bulk membrane. The greater attenuation in diffusion of DLiPC (which diffuses faster than DPPC under standard conditions due to the energetic penalty for packing of unsaturated tails) may therefore indicate a greater increase in chemical potential. Although more rigorous calculations would be required to prove this, it seems to provide a likely explanation for the changing lipid composition, namely that removal of DLiPC into the bulk membrane decreases the total Gibbs free energy by minimizing the effect of steric crowding.

**Figure 5:**
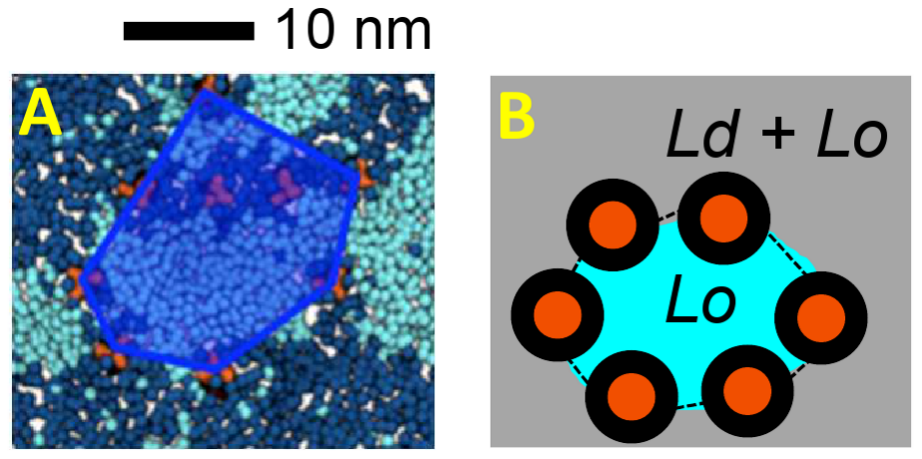
Schematic of raft-like patches of HA. **A** HA patch (blue polygon) from simulation *10HAa* showing the enrichment of Lo phase lipids (cyan) within the patch. **B** Schematic of the formation of Lo-lipid enriched nanodomains within a patch of HA proteins (orange, with lipid annuli shown in black) interacting (broken black lines) via their ectodomains.

In trying to understand the mutual interplay of HA and lipids, two key outcomes of the simulations should be considered: (i) that HA seems to prefer to occupy a position at the interface between L_o_ and L_d_ domains; and (ii) that HA TM domains do not form direct contacts, but are separated by lipids, because aggregation contacts are formed between the bulky ectodomains. To accommodate these preferences one would expect HA proteins to line up along domain boundaries and/or form nanodomains of lipids within a protein cluster. Within the protein clusters, the fraction of DPPC is ca. 65%, indicating that there is a degree of preference for Lo nanodomains within the HA clusters (Fig. 5). The clusters formed in the simulations are of dimensions ca. 10 to 20 nm. This is of interest given the suggestions that rafts in living cells may correspond to dynamic nanoassemblies of dimensions 10 to 50 nm [2].

Another interesting feature which developed during all four *10HA* simulations was a positive outward curvature of the membrane in the EC direction (Fig. 1C). The reasons for this effect are not yet clear, but are of interest given that transfected HA and NA have been shown to be sufficient for budding of virus-like particles (VLPs) from the plasma membrane [45]. This feature of the simulations will therefore be investigated further in future work.

### Subdiffusive lipid motions

A recent simulation study has shown that crowding of membranes with high concentrations of TM protein causes the transition from anomalous (subdiffusive) to normal lipid diffusion to take place over significantly longer timescales than for non-crowded membranes [46]. To consider whether our calculated diffusion coefficients (Fig. 4) may have been representative of anomalous subdiffusion, we calculated the scaling exponent associated with our calculated diffusion coefficients. The mean-square displacement of the lipids is assumed to scale as a power-law according to the relationship MSD(*t*)∼*t* ^α(*t*)^, where α(*t*) is the time-dependent scaling exponent, which can be obtained as the slope of a plot of log(MSD) against log(*t*). For normal (i.e. random walk-like) lipid diffusion, this exponent is equal to 1, whereas anomalous diffusion due to protein crowding results in α<1. We calculated the associated values of α, finding average values of 0.90 and 0.93 for DLiPC and DPPC respectively within the clusters, and 1.0 for each type of lipid in the bulk membrane.

The diffusion coefficients shown in Fig. 4 therefore arise from normal diffusion in the case of the measurements in bulk membrane, whereas those for lipids within the protein clusters arise from anomalous subdiffusion. This agrees with the findings of Javanainen *et al.* [46], who observed that normal lipid diffusion behavior is likely to occur only on timescales of micro- to milliseconds for the most crowded membranes. In the case of our simulation set-up, where lipids can exchange between the cluster and bulk membrane over much shorter timescales, it may not even be possible (even with exceptionally long simulation times) to calculate diffusion coefficients for the specific region of membrane contained within the cluster in a way that represents truly normal diffusion. However, in this study we rely on the diffusion coefficient analysis only for relative comparison of the properties of the bulk membrane and cluster interior. By measuring these diffusion coefficients in a way such that they are associated with mean displacements of ∼2 nm, this establishes a common basis for comparison. Furthermore, the diffusion coefficients measured with timesteps ranging from 75 to 100 ns (from which all of the diffusion coefficient data in Fig. 4 are drawn) display a continual decrease, with a change of less than 5% of the absolute value in all cases. Clearly this variation is likely to be greater over longer timescales, but this analysis indicates that the anomalous diffusion would not be likely to have a major effect on the qualitative trends we have observed. We also note that the enrichment of raft-type lipids within the protein clusters is manifested as a significant effect over the timescales simulated here, emphasizing the utility of comparing diffusion data measured over similar timescales. However, it is clear that the diffusion coefficients shown in Fig. 4 for lipids within the protein clusters should not be compared uncritically with other data.

## Discussion

The association of TM proteins with lipid rafts in the plasma membrane is an important factor in a wide range of membrane activities [2], including the assembly of the influenza virus [5]–[7]. However, despite the efforts of a large number of experimental studies [2], the nature and driving force for this process remain uncertain.

Experimental studies have shown that raft-associating TM proteins partition into the Ld domain in model membranes such as giant unilamellar vesicles (GUVs) [18]–[22]. The reason for this apparently paradoxical behavior may derive from a number of differences between these simplified systems and the plasma membranes they are designed to model, such as the high concentration of membrane proteins in the plasma membrane (estimated at up to 60% of membrane dry mass [23]), the presence of the actin cytoskeleton, endo-/exocytosis, lipid diversity and leaflet asymmetry. The first two factors are commonly invoked as explanations for the small size of lipid rafts in the plasma membrane, and are also thought to influence the association of membrane proteins with rafts. For example, the budding of filamentous influenza virions (but not spherical virions) has been suggested to be dependent on interactions between lipid rafts and the actin cytoskeleton [47], although the exact nature of these interactions is not yet clear. The link between high membrane protein concentration and raft-partitioning of membrane proteins has also proved difficult to elucidate. Firstly, it is generally not possible to incorporate membrane proteins in model membranes (such as GUVs) at concentrations approaching those of the plasma membrane [41]. Giant plasma membrane vesicles (GPMVs), which are extracted from live cell membranes via chemically-induced blebbing, provide a tractable model membrane which is much closer in complexity to the plasma membrane, and which is thought to include the full complement of membrane proteins [48]. Phase separation of these membranes into optically resolvable domains can be induced by reducing temperatures below ∼25°C [49]. Two studies of the partitioning of HA between these induced Lo and Ld domains have been performed, but with complex results: one study indicated partitioning into Ld domains [22], and the other indicated variable partitioning into both Ld and Lo domains, possibly reflecting compositional differences between individual GPMVs [43].

The simulations reported here thus provide a molecular level insight into the interactions of HA and lipid domains. By embedding a patch of HA proteins within a larger domain-forming membrane (i.e. a high *local* protein concentration – a difficult feature to investigate experimentally), it was possible to observe the exchange of lipids between the HA cluster and the surrounding membrane. The simulations resulted in spontaneous enrichment of raft-type lipids (DPPC and cholesterol) within the HA clusters (Fig. 2). The dimensions of the clusters (ca. 10 to 20 nm) are within the likely size-range for rafts in mammalian cells [2]. Although the cluster properties were still evolving at the end of the simulations, the main outcomes of this study are derived from the overall trend of increasing concentration of raft-type lipids, as observed in the four separate simulations. The final equilibrated state of the systems cannot easily be predicted, but complete replacement of unsaturated lipids within the cluster by raft-type lipids would be unlikely. It is useful to note that the increase in raft-type lipids in the viral membrane compared to the MDCK apical membrane (as determined by quantitative shotgun mass spectrometry) is relatively small: from 14% to 19% for sphingolipids, and from 46% to 53% for sterols [4].

With respect to the native situation, the results imply that a high local concentration of HA may be sufficient as a driving factor for the association of the protein with lipid rafts in the plasma membrane. This hypothesis is supported by experiments showing that cross-linking of HA with antibodies or cholera toxin subunit B induces spatial correlation of the protein with other raft markers in plasma membranes over long length scales, suggesting that oligomerization of raft components can increase raft size [50], [51]. While highly concentrated clusters of HA have been directly visualized in the plasma membrane using immunogold-labeling EM and FPALM [6], [13], [14], this gives rise to the question of which processes may drive HA clustering.

A limitation of the current study should be noted, namely that *in vivo* the HA ectodomain is glycosylated at a number of sites, which may alter its propensity for aggregation. The degree of direct contact between HA proteins observed in the simulations may thus not be fully representative of the situation in plasma membranes. However, EM images of plasma membranes appear to show HA clusters of similarly high concentrations [6], and in hexagonally packed clusters [13], for which direct protein-protein contacts are a possible explanation. Intracellular domain contacts have also been suggested to mediate targeting of the linker for activation of T cells (LAT) to rafts [21]. A simulation study of HA fragments, comprising only the TM and cytoplasmic regions, would be a useful approach to understanding the influence of protein-protein contacts on the behavior observed here. Aggregation state affects the diffusion coefficient of TM proteins [52], and so could be expected to have a possible influence on the lipid domain dynamics observed here. Such a study would also be of relevance to experimental investigations of similar fragments of HA [12], [53], and the TM peptide of LAT [20], [21]. It is clear, however, that protein-protein contacts are not solely responsible for HA clustering in the plasma membrane. For example, a non-raft-associating mutant of HA (with alterations in the TM region) was shown to be randomly distributed in the plasma membrane, and resulted in viruses with reduced HA incorporation and infectivity [6].

Another potential explanation for HA clustering is that enrichment of raft-type lipids stabilizes areas of high HA concentration. Together with our findings, this would imply a type of positive feedback mechanism, in which high local HA concentration drives association with raft-type lipids, and high concentrations of raft-type lipids help to stabilize HA clusters. The simulations did appear to indicate an ability of lipid domains to influence the aggregative behavior of HA, in that regions of unsaturated lipid in some cases appeared to cause proteins to move apart. A complete explanation of this effect would require a separate in-depth study. However, this view would seem to be supported by the cross-linking studies indicating strengthened raft-association upon cross-linking of HA in live cell membranes [50], [51].

A variety of experimental and simulation studies have investigated other factors which can affect protein aggregation in biological and model membranes in general, such as the degree of hydrophobic mismatch between protein and lipid [36], [52], [54]–[58] (which can also affect local stretching or compression of the surrounding membrane [59], [60]), membrane curvature [52], [61], [62], and the effect of cholesterol on adaptations to the protein-lipid interface [57]. It is also feasible that the degree of hydration at the membrane-water-protein interface (a function of the TM region structure, as well as the membrane environment [63]) may affect protein aggregation.

The HA sequence contains two important raft-targeting signals: a group of three acylated cysteines, and a group of three consecutive hydrophobic residues at the N-terminal end of the TM region (530–532 in the HA isoform simulated here). Of the acylated cysteines, two are located in the cytoplasmic tail and palmitoylated, whereas the third residue located in the TM region is thought to be specifically modified with a stearic acid (18-carbon, saturated) [64]. Palmitoylation has been suggested to regulate raft-association for the majority of integral raft proteins, as shown in a study using GPMVs, which phase separate into two large ordered and disordered domains [65]. However, while most integral raft proteins were found to partition into the ordered phase, HA and a number of other raft-associating proteins failed to enrich in the ordered phase. The importance of interactions with the cytoskeleton, which is not present in GPMVs, is one possible explanation. Mutation of the acylated cysteines has been shown to prevent incorporation of HA into detergent-resistant membranes [66], [67] and abolish FRET between HA and raft markers in live cells [11], [12]. However, non-palmitoylated mutants have a varied effect on viral replication, depending on the specific viral strain and type of host cell [67], [68]. In the case of our simulations, the palmitoyl chains appear to cause the formation of an annulus of saturated lipids in the IC leaflet. In conjunction with the DLiPC-enriched EC leaflet annulus, this results in domain antiregistration in the area local to the protein. It seems probable that the differing compositions of the annuli in each leaflet may be an important driving force in the partitioning of HA at domain boundaries. A recent simulation study indicated that palmitoylation causes the WALP TM peptide to partition at domain boundaries in similar ternary mixture bilayers [39].

The three hydrophobic residues 530–532 are situated at the level of the EC leaflet lipid phosphates in our simulations. A number of experimental studies have indicated the importance of these residues for raft-targeting of HA [69]. EM images of immungold-stained HA in the plasma membrane of MDCK cells displayed concentrated clusters of wildtype HA, while a 530–532 alanine mutant was distributed randomly [6]. Another study employed fluorescence recovery after photobleaching (FRAP) to measure the lateral diffusion of HA in live cells, indicating a cholesterol-dependent increase in mutant HA diffusion coefficient compared to wildtype [70]. A recent study indicated that the same residues are required for FRET between mutant HA and raft markers in live cells [11]. Two mutations in the same region have also been reported to abolish ordering of lipids in proximity to a TM fragment of HA, as measured by electron spin resonance [53]. The underlying mechanism by which the hydrophobic residues 530–532 target HA to lipid rafts is unclear. One possibility is that they affect the hydrophobic mismatch between the protein and its surrounding membrane environment. This factor has been shown to have an effect on both TM protein aggregation [36], [52], [54]–[58] and lipid domain interactions [35]. However, hydrophobic matching is unlikely to be the sole determinant of raft-association in TM proteins [65], [71]. It would be of interest to conduct a detailed CG simulation study of the specific effects of both HA acylation and mutations of the hydrophobic residues 530–532.

An important feature of the *in vivo* plasma membrane is the pronounced compositional asymmetry between the outer and inner leaflets of the lipid bilayer. Thus, sphingolipids and phosphatidylcholine lipids are enriched in the EC leaflet, while the IC leaflet is enriched in phosphatidylserine and phosphatidylethanolamine, and carries a net negative charge [72]. Compared to the compositionally symmetric membrane systems simulated here, these features would likely have an effect on the interactions of HA with lipid domains. For example, the finding that a HA TM fragment induces lipid ordering via interactions with negatively charged lipids may be relevant [53]. However, it is difficult to make predictions for such complex systems. The membranes studied here allow for a more direct comparison with experiments conducted with compositionally symmetric model membranes. Other features of plasma membranes absent from model membranes include a great degree of lipid diversity, endo-/exocytic processes, and cytoskeletal interactions. The inclusion of these aspects of complexity in simulations of biological membranes will be a challenge for future research in this area.

Overall, it seems clear that a number of different competing processes, arising from the various interactions between proteins, lipids, and the cytoskeleton, are likely to contribute to the formation of rafts in the plasma membrane, and their association with membrane proteins. This highlights the importance of studying relatively simple systems which allow for the isolated investigation of individual processes, such as the influence of high local protein concentration. The diversity of causal factors may also go some way to explaining the conflicting behavior observed in some experiments. For example, cholesterol depletion by methyl-β-cyclodextrin (which is thought to disrupt lipid rafts) has been shown to reduce FRET between HA and raft markers expressed in Chinese hamster ovary cells [11], [12], and results in extensive structural defects in virions released from Madine-Darby canine kidney cells, leading to reduced infectivity [73]. Conversely, HA clusters observed in fibroblast plasma membranes by immunogold-labeling EM were unaffected by treatment with methyl-β-cyclodextrin or glycosphingolipid synthesis inhibitors [13]. It is also possible that rafts may exist in a range of different forms, as suggested by the recent finding that GPMVs can be induced to form domains of varying properties, depending on the method of extraction [74].

Following the demonstration that the MARTINI CG forcefield was able to reproduce the properties of domain-forming membranes [34], a number of recent studies have built upon this finding and investigated how TM proteins interact with these domains. The first showed that model α-helical TM peptides partition into Ld domains [36], as expected from previous experimental studies [18], [55]. The second showed that extreme crowding of a membrane with such peptides could induce lipid domain formation in membranes which would otherwise be mixed, with the peptides partitioning into the Ld domains [37]. More recently, a study of a range of TM and peripheral membrane proteins has indicated that palmitoylation of WALP causes it to associate with domain boundaries, whereas the doubly palmitoylated LAT TM peptide was found to partition into the Ld domain [39]. Our results indicate that more complex, cell-like behavior – the formation of nanoassemblies enriched in raft-type lipids – may be observed if the system is set up to allow exchange of lipids between an area of high local protein concentration and the surrounding membrane, and when protein-protein interactions beyond the immediate bilayer region are included in the simulation model.

## Methods

### HA model

The model of HA (Fig. 1) was based on the X-ray structure (PDB code: 1MQM) of the protein from the A/duck/Ukraine/1/63 (H3N8) influenza strain [75], which was converted to the CG representation using the standard MARTINI tools [31], [32]. The X-ray structure includes the ectodomain, but excludes the TM and cytoplasmic domains, and a short linker between the ectodomain and TM domain. This missing sequence was modeled as α-helix and added to the ectodomain crystal structure; palmitoyl chains were added at the three sites towards the IC side of the TM domain (Cys555, Cys562 and Cys565; see Supporting Information for further details). The model of the intact HA trimer was then simulated in a bilayer patch, allowing for relaxation of the added structure. A clustering algorithm was then used to select the most representative conformation. To maintain protein structure, elastic network restraints were applied with the ElNeDyn tool [76], using a cutoff of 1.4 nm and force constants of 1000 kJ mol^−1^ nm^−2^. The cytoplasmic domain was treated as unstructured and excluded from the restraint network. The TM domain sequence is ^530^WILWISFAISCLLLCVVLLGFIMWACQ^556^. For further details of this and other Methods please see Supporting Information Text S1.

### Simulation set-up

Bilayers were formed using packmol [77], as described in the Supporting Information. The 50×50 nm^2^ bilayers contained 3706 DPPC, 3706 DLiPC and 3120 cholesterol molecules (a ratio of 0.35 : 0.35 : 0.3 respectively), with equal proportions in either leaflet. HA proteins were arranged in a regular array and inserted into the bilayers using the Gromacs g_membed tool [78]. The average lateral spacing between the centers of mass of adjacent proteins was 7 nm, and the average minimum distance between adjacent protein surfaces was 2 nm. The resulting systems were solvated with MARTINI CG water, including sufficient Na^+^ ions to neutralize the 9 negative charges present on each HA molecule, and 5% “antifreeze” particles, as detailed in the original MARTINI paper [31]. Systems were energy minimized for ∼500 steps of the steepest descents algorithm, and briefly equilibrated for 100 MD steps at 323K. The first *10HA* production simulation (labeled *a* in Figs. 2 and 3) was run for 4 µs at 310 K, then for 8 µs at 295 K. The *1HA-DLiPC* production simulation was run for 8 µs at 295K. All other production simulations were run for ∼12 µs at 295K (equivalent to the temperature used in the original MARTINI study of domain formation [34].

### Simulation details

All simulations were conducted using the Gromacs package (www.gromacs.org) [79] and the MARTINI CG force field [31], [32]. The integration time step was 10 fs. Lennard-Jones and Coulomb interactions were shifted to zero between 0.9 and 1.2 nm, and 0 and 1.2 nm respectively. The Berendsen thermostat [80] (coupling constant of 1.0 ps) and barostat (coupling constant of 1.1 ps; compressibility of 1.0×10^−6^ bar^−1^; reference pressure of 1 bar) were used. Visualization was performed with VMD [81], and analysis with the MDAnalysis Python library [82]. All reported simulation times and time-dependent data have been adjusted to account for the faster sampling of the MARTINI model; times have thus been multiplied by a factor of 4. The comparison of time-dependent data to experimental work should be considered semi-quantitative.

## Supporting Information

Figure S1 Analysis of one of the two *1HA* simulations. a) Lipid lateral diffusion coefficients, with respect to distance from the HA protein, analyzed over the final 4 µs of the simulation. Lipid displacements were measured within concentric rings of width 1 nm, radiating out from the geometric center of the protein TM domain. b) Lipid tail ordering, represented by the average of the angles between the two terminal lipid tail bonds (those between the second and third CG tail particles, and the third and fourth) and the bilayer normal. Opaque lines show the angles averaged over the last 4 µs, and transparent lines show ± 1 mean deviation.
(PDF)

Figure S2 Lateral paths of the ten proteins in the *10HAb* simulation. The starting position of each protein is indicated with a black dot, and their subsequent paths in the plane of the membrane are shown in different colors.
(PDF)

Figure S3 Final configuration of the *0HA* simulation: a 50×50 nm^2^ bilayer of the same ternary lipid composition as the *10HA* simulations. DPPC phosphates are shown in light blue, DLiPC phosphates in dark blue, and cholesterol alcohol headgroups (mostly hidden beneath the PC phosphates) in green. Only the EC leaflet is visible, but the distribution of domains in the IC leaflet is essentially the same. Application of periodic boundary conditions indicates that the system represents a single large Ld domain, surrounded by a continuous Lo domain.
(PDF)

Figure S4 Percentage of cholesterol within the protein aggregate during the *10HA* simulations. Details of the algorithm for defining the cluster interior are included in Text S1. Analysis of individual leaflets was not conducted, due to flip-flop of cholesterol between leaflets.
(PDF)

Figure S5 Analysis of the *1HA-DLiPC* simulation: a) lipid diffusion coefficients, and b) tail ordering. The analyses were conducted as described in the caption of Fig. S1. In the tail order analysis, opaque lines show the angles averaged over the last 4 µs, and transparent lines show ± 1 mean deviation.
(PDF)

Figure S6 Snapshots of the final configurations of the four *10HA* simulations (*a–d*), overlaid with outlines of the cluster interiors, determined according to the algorithm described in Text S1. This algorithm was used to define the areas of membrane analyzed in Figs. 3 and 4, and Fig. S4. Only the EC leaflets are shown here.
(PDF)

Figure S7 Percentage of DPPC within the protein aggregate during the *10HA* simulations, analyzed with three different cutoffs for the algorithm for defining the cluster interior. The details of the algorithm are included in Text S1.
(PDF)

Text S1 Supporting text is available, containing further details related to the HA model, and the set-up of the bilayer systems. The algorithm for definition of the cluster interior is also described in detail.
(PDF)
