# Supplementary figures and images for: Formation of Raft-Like Assemblies within Clusters of Influenza Hemagglutinin Observed by MD Simulations
Source: PLoS Comput Biol. 2013 Apr 11;9(4):e1003034. doi: 10.1371/journal.pcbi.1003034 (PMC3623702; doi:10.1371/journal.pcbi.1003034)

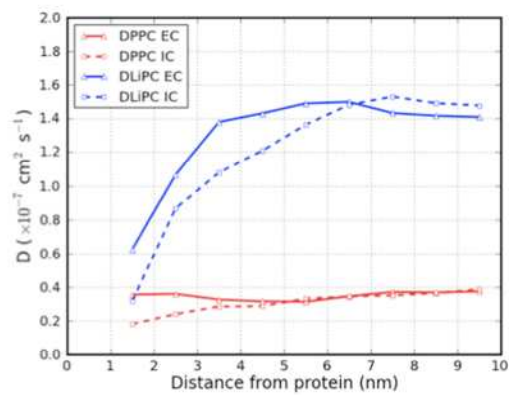

a)

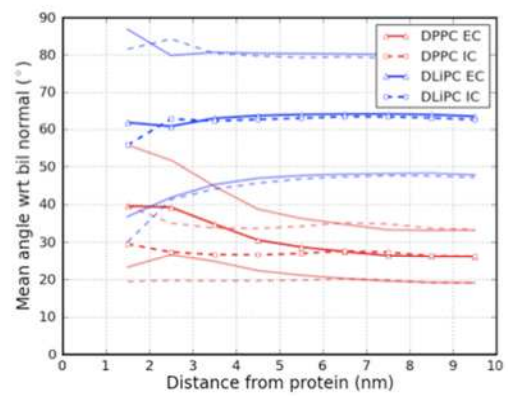

b)

Supplement: Figure S1 — Analysis of one of the two 1HA simulations. a) Lipid lateral diffusion coefficients, with respect to distance from the HA protein, analyzed over the final 4 µs of the simulation. Lipid displacements were measured within concentric rings of width 1 nm, radiating out from the geometric center of the protein TM domain. b) Lipid tail ordering, represented by the average of the angles between the two terminal lipid tail bonds (those between the second and third CG tail particles, and the third and fourth) and the bilayer normal. Opaque lines show the angles averaged over the last 4 µs, and transparent lines show ± 1 mean deviation. (PDF) [file pcbi.1003034.s001.pdf]

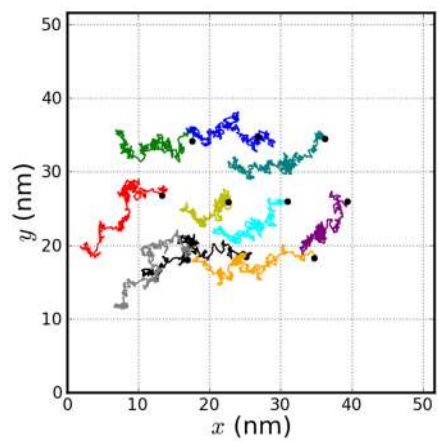

Supplement: Figure S2 — Lateral paths of the ten proteins in the 10HAb simulation. The starting position of each protein is indicated with a black dot, and their subsequent paths in the plane of the membrane are shown in different colors. (PDF) [file pcbi.1003034.s002.pdf]

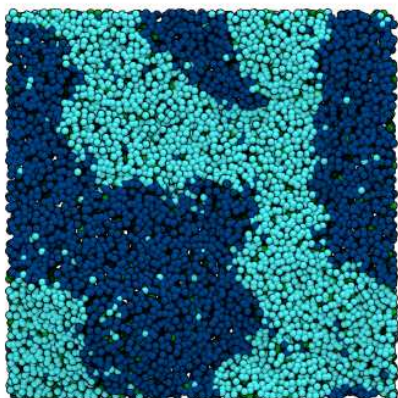

Supplement: Figure S3 — Final configuration of the 0HA simulation: a 50×50 nm2 bilayer of the same ternary lipid composition as the 10HA simulations. DPPC phosphates are shown in light blue, DLiPC phosphates in dark blue, and cholesterol alcohol headgroups (mostly hidden beneath the PC phosphates) in green. Only the EC leaflet is visible, but the distribution of domains in the IC leaflet is essentially the same. Application of periodic boundary conditions indicates that the system represents a single large Ld domain, surrounded by a continuous Lo domain. (PDF) [file pcbi.1003034.s003.pdf]

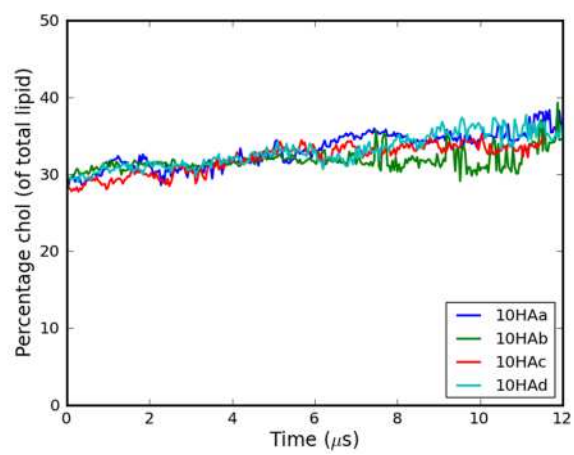

Supplement: Figure S4 — Percentage of cholesterol within the protein aggregate during the 10HA simulations. Details of the algorithm for defining the cluster interior are included in Text S1. Analysis of individual leaflets was not conducted, due to flip-flop of cholesterol between leaflets. (PDF) [file pcbi.1003034.s004.pdf]

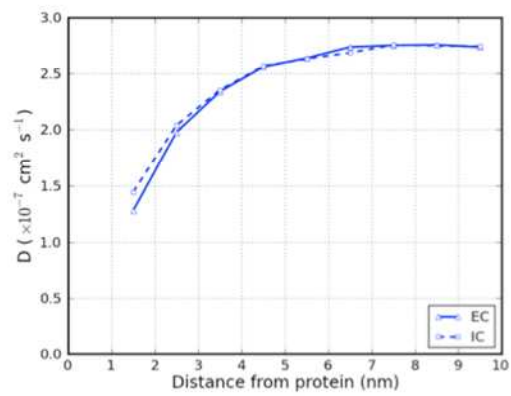

a)

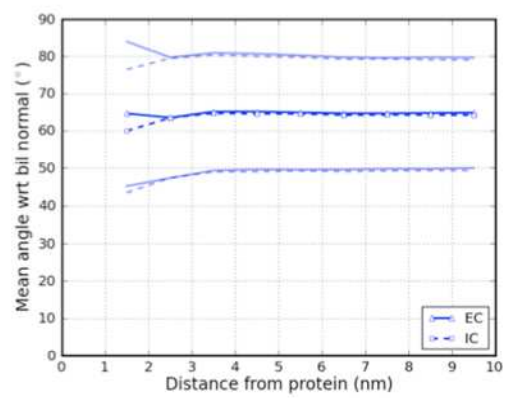

b)

Supplement: Figure S5 — Analysis of the 1HA-DLiPC simulation: a) lipid diffusion coefficients, and b) tail ordering. The analyses were conducted as described in the caption of Fig. S1. In the tail order analysis, opaque lines show the angles averaged over the last 4 µs, and transparent lines show ± 1 mean deviation. (PDF) [file pcbi.1003034.s005.pdf]

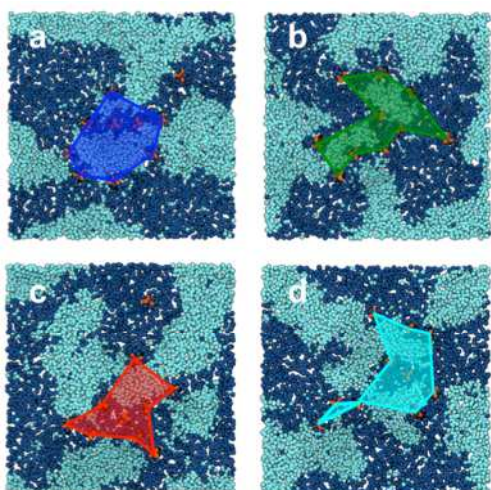

Supplement: Figure S6 — Snapshots of the final configurations of the four 10HA simulations (a–d), overlaid with outlines of the cluster interiors, determined according to the algorithm described in Text S1. This algorithm was used to define the areas of membrane analyzed in Figs. 3 and 4, and Fig. S4. Only the EC leaflets are shown here. (PDF) [file pcbi.1003034.s006.pdf]

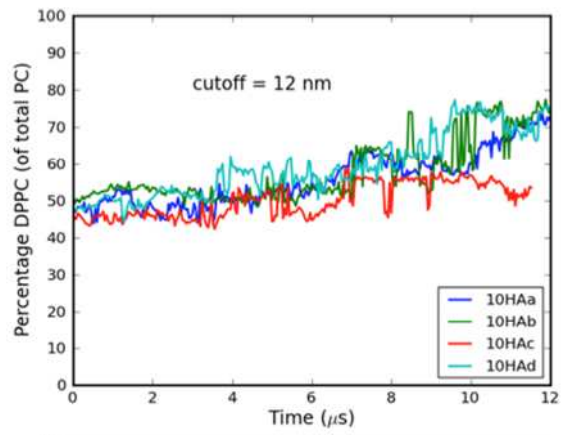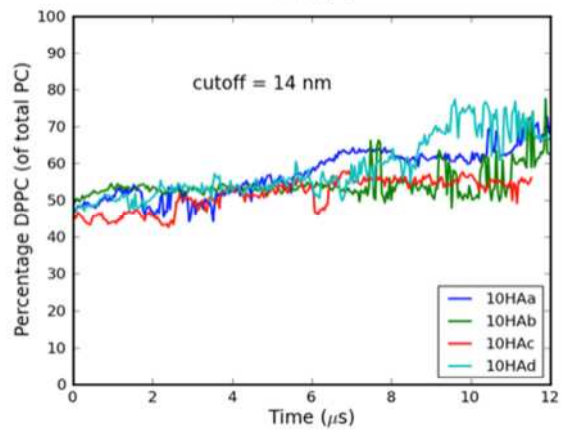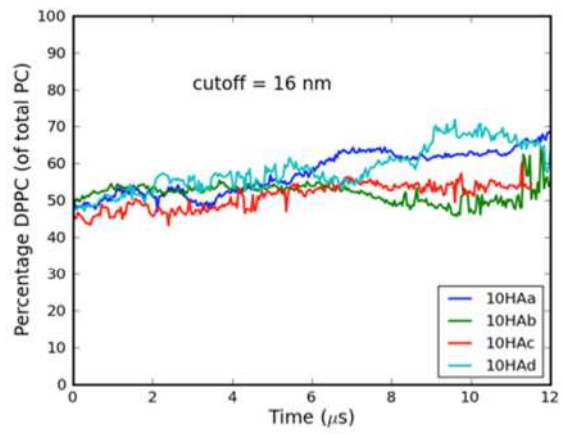

Supplement: Figure S7 — Percentage of DPPC within the protein aggregate during the 10HA simulations, analyzed with three different cutoffs for the algorithm for defining the cluster interior. The details of the algorithm are included in Text S1. (PDF) [file pcbi.1003034.s007.pdf]
