# Supplementary material for: Formation of Raft-Like Assemblies within Clusters of Influenza Hemagglutinin Observed by MD Simulations
Source: PLoS Comput Biol. 2013 Apr 11;9(4):e1003034. doi: 10.1371/journal.pcbi.1003034 (PMC3623702; doi:10.1371/journal.pcbi.1003034)
Supplement: Text S1 — Supporting text is available, containing further details related to the HA model, and the set-up of the bilayer systems. The algorithm for definition of the cluster interior is also described in detail. (PDF) [file pcbi.1003034.s008.pdf]

Supporting Information: Text S1

Formation of Raft-Like Assemblies within Clusters of Influenza Hemagglutinin Observed by MD Simulations

Daniel L. Parton, Alex Tek, Marc Baaden, Mark S. P. Sansom

Extended Methods and Materials

HA Model. The sequence missing from the crystal structure comprised a twelve-residue linker, the TM domain, and a ten-residue cytoplasmic domain. The linker and TM domain were modeled as an ideal α-helix and converted to CG representation using the MARTINI tools [1,2], as for the ectodomain. The last five residues from the C-terminal end of the crystal structure were also included in the modeled α-helix, which thus comprised residues 518-556. This allowed the helix to be aligned with the same residues in the crystal structure. A bend in the helix was then required to orient the TM domain downwards toward the membrane. This was achieved by modeling the helix with a simple elastic network model, then adding harmonic restraints to bend the helix into the correct orientation, with rigid constraints to retain ideal α-helical structure in the TM domain.

The process was repeated for all three of the protein subunits, and the resulting structure was inserted into a pre-formed 15 × 15 nm^2 CG bilayer (comprising 0.35 : 0.35 : 0.3 DPPC/DLiPC/chol, and formed using the same process as described below for other bilayers) using g_membed [3]. The initial position of the protein in the z-axis (equivalent to the bilayer normal) was determined by matching the central TM domain residue (Leu543) to the bilayer center. System parameters were created using the MARTINI CG force field [1,2], with additional "H-bond" type restraints (which link backbone particles four residues apart) applied to the TM domain and linker, and elastic network model (ENM) restraints applied to the ectodomain with a cutoff of 1.4 nm (the latter using the ElNeDyn tool [4]). Force constants of 1000 kJ mol^{-1} nm^2 were used for all restraints. The system was energy minimized using ~ 500 steps of the steepest descents algorithm, then simulated with MD for 4 μs. The use of H-bond restraints allowed the TM domain and linker to flex and rotate without deviating from an overall α-helical structure. During the simulation, the helices coiled to a small degree, allowing the four polar residues within the TM domain (S535, S539, C540 and C544), which were positioned on the same face of the ideal α-helix, to be buried away from the hydrophobic lipid tails. The last 2 μs of the simulation were clustered using the single-linkage algorithm, to find the most representative structure.

The cytoplasmic tail plus seven residues of the TM domain (residues 550-566) was initially modeled as an ideal α-helix and added to the simulated TM domain structure by aligning the helix backbone particles. In subsequent simulations, restraints were not added to the cytoplasmic tail itself, and coil-type parameters were applied, reflecting the largely unstructured nature of the cytoplasmic region. Palmitoyl chains were then added at the appropriate cysteines, initially oriented perpendicularly from the helix backbone. The chains each comprised four CG particles, with the same parameters as the palmitoyl chains of MARTINI DPPC. A similar parameterization has been used very recently in simulations of rhodopsin [5]. The complete protein structure was again inserted into a preformed 15 × 15 nm^2 CG bilayer; the system was energy minimized and simulated three times, each for 4 μs (with different randomly generated starting velocities), with ENM restraints on the ectodomain, H-bond restraints on the linker and TM domain, and no restraints on the

cytoplasmic tail. In all simulations, the cytoplasmic tail remained mostly associated at the membrane interface, allowing the attached palmitoyl chains to insert into the IC leaflet of the bilayer. The HA structure from the final snapshot of one of these simulations was used in all subsequent simulations.

Bilayer Modeling. The 20 × 20 nm^2 bilayers used in simulations with a single HA protein were initially formed using the Packmol software [6], which allowed copies of individual lipid molecules to be packed within the necessary spatial constraints. This method also allowed control over the lipid composition in each bilayer leaflet. The DLiPC bilayer comprised 1024 lipids. The domain-forming bilayer comprised 560 DPPC molecules, 560 DLiPC molecules and 480 chol molecules (a ratio of 0.35: 0.35 : 0.3). The bilayers were each energy minimized with ~ 500 steps of the steepest descents algorithm, then equilibrated with a 400 ns CG simulation at 323K. In the case of the domain-forming bilayer, this temperature was useful in preventing large-scale domain formation from occurring prior to protein insertion.

The larger, 50 × 50 nm^2 domain-forming membranes were each constructed by tiling a pre-formed 12 × 12 nm^2 bilayer, which was itself assembled and equilibrated in the same way as described for the 20 × 20 nm^2 bilayer. This process was conducted for each of the four membrane patches used in the *10HA* simulations, with different randomizing seeds for the initial packing carried out with Packmol.

Algorithm for Definition of the Cluster Interior

The areas of membrane included in the analysis in Figs. 3 and 4, and SI Fig. S4 were defined by drawing triangles between each the center of each protein TM domain and taking their combined area, while ignoring triangles with any side longer than 14 nm. The latter part of the algorithm was used to exclude the small number of lipids which were technically within the cluster but more than ~ 7 nm from a protein. This distance was chosen from analysis of the *1HA* and *1HA-DLiPC* simulations, which showed that the protein had no effect on lipid diffusion rates or tail ordering at a distance of 7 nm (SI Figs. S1 and S5). Snapshots overlaid with outlines of the analyzed membrane areas are shown in SI Fig. S6. The analysis was repeated with two different values for the cutoff (one lower, at 12nm, and one higher, at 16 nm). This analysis suggested that the exact value of the cutoff this choice did not significantly change the overall finding of an increase in DPPC concentration (SI Fig. S7). While the choice of cutoff can significantly influence the choice of cluster for an individual simulation, the average behavior in each case appears to be an increase in DPPC concentration. The increase in DPPC was a little less apparent for the largest cutoff of 16 nm, confirming our initial choice of a more conservative value of 14 nm.

In the analysis of individual leaflets (in Fig. 3 of the main text), lipids within 2 nm of a protein (i.e. the lipid annuli) were ignored. The lipid composition of these regions remained constant throughout the simulations, and the analysis including these regions differed only by a small constant shift to the data. Ignoring the lipid annuli allowed for analysis of only the regions of the cluster interior which changed significantly over time.

References

1. Marrink SJ, Risselada HJ, Yefimov S, Tieleman DP, Vries AHd (2007) The MARTINI force field: coarse grained model for biomolecular simulations. J Phys Chem B 111: 7812-7824.
2. Monticelli L, Kandasamy SK, Periole X, Larson RG, Tieleman DP, et al. (2008) The MARTINI Coarse-Grained Force Field: Extension to Proteins. J Chem Theory Comput 4: 819-834.
3. Wolf MG, Hoefling M, Aponte-Santamaría C, Grubmüller H, Groenhof G (2010) g_membed: Efficient insertion of a membrane protein into an equilibrated lipid bilayer with minimal perturbation. J Comput Chem 31: 2169-2174.
4. Periole, Cavalli, Marrink, Ceruso (2009) Combining an Elastic Network With a Coarse-Grained Molecular Force Field: Structure, Dynamics, and Intermolecular Recognition. J Chem Theory Comput 5: 2531-2543.
5. Baoukina S, Marrink SJ, Tieleman DP (2012) Molecular Structure of Membrane Tethers. Biophys J 102: 1866-1871.
6. Martínez L, Andrade R, Birgin EG, Martínez JM (2009) PACKMOL: a package for building initial configurations for molecular dynamics simulations. J Comput Chem 30: 2157-2164.
